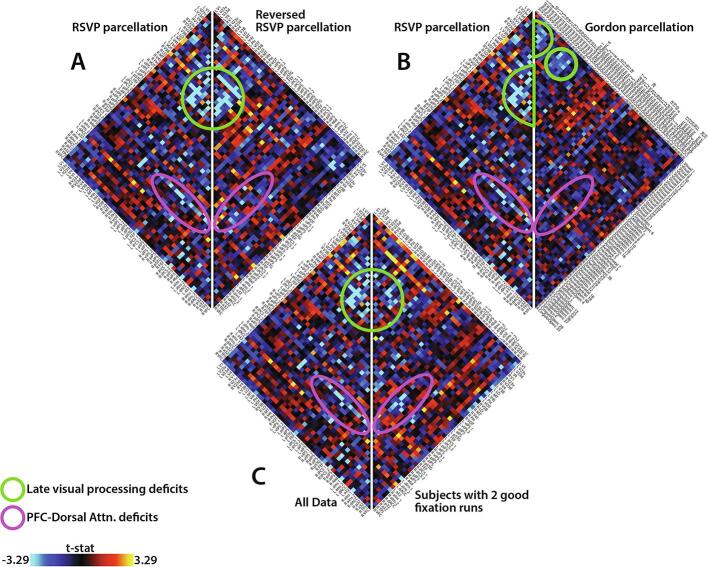# Deficits and compensation: Attentional control cortical networks in schizophrenia

**DOI:** 10.1016/j.nicl.2020.102348

**Published:** 2020-07-20

**Authors:** Sophie C. Arkin, Daniel Ruiz-Betancourt, Emery C. Jamerson, Roland T. Smith, Nicole E. Strauss, Casimir C. Klim, Daniel C. Javitt, Gaurav H. Patel

**Affiliations:** a University of California, Los Angeles 90095, United States; b Columbia University Irving Medical Center, 10032, United States; c Hunter College, 10065, United States; d Palo Alto University, 94301, United States; e University of Michigan Medical School, 48109, United States; f Nathan Kline Institute, 10962, United States; g New York State Psychiatric Institute, 10032, United States

**Keywords:** Dorsal attention network, Ventral attention network, Resting state functional connectivity, fMRI, Temporal parietal junction, Prefrontal cortex

## Abstract

Visual processing and attention deficits are responsible for a substantial portion of the disability caused by schizophrenia, but the source of these deficits remains unclear. In 35 schizophrenia patients (SzP) and 34 healthy controls (HC), we used a rapid serial visual presentation (RSVP) visual search task designed to activate/deactivate the cortical components of the attentional control system (i.e. the dorsal and ventral attention networks, lateral prefrontal regions in the frontoparietal network, and cingulo-opercular/salience networks), along with resting state functional connectivity, to examine the integrity of these components. While we find that behavioral performance and activation/deactivation of the RSVP task are largely similar between groups, SzP exhibited decreased functional connectivity within late visual components and between prefrontal and other components. We also find that performance correlates with the deactivation of the ventral attention network in SzP only. This relationship is mediated by the functional connectivity of critical components of the attentional control system. In summary, our results suggest that the attentional control system is potentially used to compensate for visual cortex deficits. Furthermore, prefrontal deficits in SzP may interfere with this compensatory use of the attentional control system. In addition to highlighting focal deficits and potential compensatory mechanisms in visual processing and attention, our findings point to the attentional control system as a potential target for rehabilitation and neuromodulation-based treatments for visual processing deficits in SzP.

## Highlights

• Examined attention systems in SzP with resting-state connectivity and task fMRI.
• SzP have functional connectivity deficits in late visual cortex and prefrontal areas.
• Task performance correlated with ventral attention network deactivation in SzP only.
• This relationship is mediated by connectivity of key attentional control components.
• Results reveal deficits and potential compensation in SzP visual processing/attention.

## 1 Introduction

Deficits in attentional control are considered to be a core feature of schizophrenia and a key contributor to cognitive dysfunction (Luck and Gold, 2008, Nuechterlein et al., 2009). Two major components of attentional control are selective attention and cognitive control. Selective attention is often measured by the ability to select target stimuli from amongst distracting stimuli during visual search. Cognitive control is often measured by the ability to inhibit automated responses during continuous performance tests (CPT), such as the CPT- Identical Pairs (CPT-IP) Attention/Vigilance test in the Measurement and Treatment Research to Improve Cognition in Schizophrenia (MATRICS) Cognitive Consensus Battery (MCCB). While cognitive control is reliably impaired in schizophrenia patients (SzP) (Nuechterlein et al., 2008), selective attention may be paradoxically intact (Gold et al., 2009). However, other studies point to attentional deficits originating in the visual system (Javitt, 2009, Martinez et al., 2012a). In the present study, we examine all of the components of the attentional control system simultaneously to identify potential deficits in SzP. Specifically, we used an fMRI visual search task designed to localize and examine the brain areas involved in various aspects of attention control (selective attention, cognitive control, and visual processing), and then used resting state functional connectivity to examine the integrity of the underlying brain networks.

**Fig. 1:**
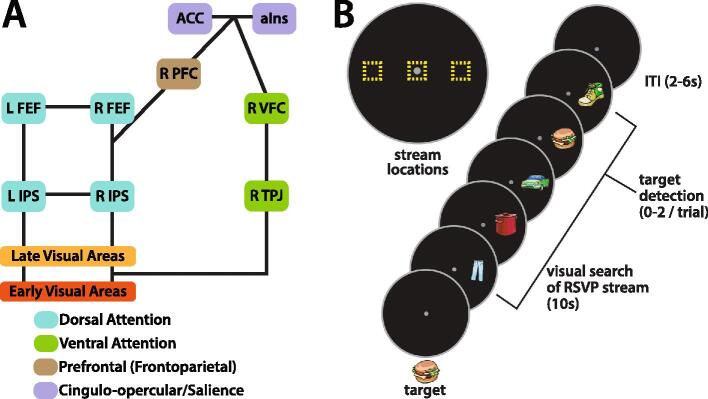
A) Corbetta-Shulman model of the attentional control system. Incoming visual information is processed first through early visual areas (yellow) designed to extract low-level visual features, then through higher-level visual areas (orange) that extract shape and object information. Dorsal attention areas (cyan) interact with these visual areas to enhance the processing of behaviorally relevant features or objects, known as top-down or goal-directed orienting of attention. While dorsal attention areas are activated, ventral attention areas (green) are deactivated. The degree of deactivation is associated with engagement of the attentional control system: increased deactivation of the ventral attention network is associated with both successful detection of targets amongst distractors in a rapid serial visual presentation (RSVP) visual search task and increased working memory load (Shulman et al., 2007, Todd and Marois, 2004). When a novel behaviorally relevant stimulus is presented, the ventral attention network spikes in activity, alerting the dorsal attention network to shift its focus. This interaction is controlled by the combination of prefrontal areas (tan) and the cingulo-opercular/salience network (purple), which maintains and updates task demands to achieve the current behavioral goals. B) RSVP task schematic: Participants viewed a target stimulus prior to start of task. During the visual search phase, participants were instructed to fixate on a centered white dot while covertly attending to the RSVP stream of clip art images in one of three locations (represented by yellow squares). Once a target stimulus was detected, participants were instructed to quickly respond by pressing a button with their index finger on a fiber optic button box. (see Supplemental Methods for detailed description). Abbreviations: *TPJ:* temporoparietal junction *IPS:* intraparietal sulcus *FEF:* frontal eye-fields *PFC:* prefrontal cortex *VFC:* ventral frontal cortex *aIns:* anterior insula *ACC:* anterior cingulate cortex. (For interpretation of the references to colour in this figure legend, the reader is referred to the web version of this article.)

Numerous neuroimaging studies have described cortical areas spanning frontal, parietal, and temporal regions that are involved in the control of visual attention. One influential model, proposed by Corbetta and Shulman, describes how these areas interact (Corbetta et al., 2008, Corbetta and Shulman, 2002) based on well-characterized macaque models of attentional and oculomotor control (Fecteau and Munoz, 2006, Lappi, 2016, Patel et al., 2015) (Fig. 1). The model subdivides these areas into component networks (hereafter called components). The core components of this model are the dorsal and ventral attention networks, which are implicated in selective attention. The dorsal attention network consists of a set of frontoparietal areas in both hemispheres, including ones along the intraparietal sulcus (IPS) and the frontal eye-fields (FEF). The ventral attention network is lateralized to the right hemisphere and consists of the temporoparietal junction (TPJ) and ventral frontal cortex (VFC). These networks interact with early (V1-V4) and late (MT, LO, and others in lateral occipital cortex) visual processing areas in the occipital lobe to enhance the processing of visual features or spatial locations that may be of interest. The functioning of these components is controlled by lateral prefrontal cortical areas within the frontoparietal and the cingulo-opercular/salience network, involved in adaptive and stable cognitive control, respectively. The cingulo-opercular/salience network includes the dorsal anterior cingulate (dACC) and anterior insula (aIns) (Dosenbach et al., 2008).

While in general, fMRI studies examining cognitive deficits have demonstrated that SzP have prominent deficits in prefrontal (Kahn et al., 2015, Goldman-Rakic and Selemon, 1997, Minzenberg et al., 2009) and visual cortex (Javitt and Freedman, 2015, Javitt, 2009), whether and how these deficits impact attentional control remains unclear. For example, one recent pair of fMRI studies of attention systems (one using a visual search task and another an oddball paradigm) in SzP revealed deficient activation of the dorsal, ventral, and cingulo-opercular/salience networks (Jimenez et al., 2016, Wynn et al., 2015). However, an earlier fMRI study examining how perceptions of spatial frequency affect visual attention in SzP found intact dorsal attention areas and impaired visual cortex functioning (Martinez et al., 2012a).

Recent studies have also used resting state functional connectivity to examine the connectivity within or between these components in SzP. Resting state functional connectivity is thought to measure a combination of anatomical connectivity and experience-dependent plasticity (Buckner et al., 2013) and is largely independent of cognitive state (Gratton et al., 2018). As with the task fMRI literature, resting state functional connectivity studies have shown deficits in many of the relevant subcomponents with no consensus. Specifically, some studies found reductions in connectivity within one component (i.e., dorsal attention network (Woodward et al., 2011), frontoparietal network (Baker et al., 2019, Baker et al., 2013, Mamah et al., 2013), while others have revealed that the notable deficits occur between components (i.e., between cingulo-opercular and salience network (Mamah et al., 2013, van de Ven et al., 2017). Another study found no group differences within or between the frontoparietal or cingulo-opercular components (Sheffield et al., 2015).

Due to the inconsistent findings in task and resting state fMRI studies about the integrity of the brain areas and networks involved in the control of attention, we hypothesized that there may be multiple deficits within and between these components —spanning prefrontal, parietal, and visual cortex—that summed together may explain impaired visual attention in SzP. In order to examine the functioning and connectivity of these components, we measured BOLD-fMRI signals evoked by the rapid serial visual presentation (RSVP) visual search task in SzP and healthy controls (HC). The RVSP task was developed by Patel and colleagues to separate BOLD activity of the cortical components involved in the attentional control system, as proposed by Corbetta/Shulman (Patel et al., 2015, Patel et al., 2010, Shulman et al., 2003). In this task, participants are instructed to detect a target stimulus while covertly attending to a rapid stream of clipart images displayed to the left, right, or superimposed over a central fixation point. During the visual search phase of the RSVP task, the areas within the visual processing, dorsal attention network, prefrontal, and cingulo-opercular/salience components are activated by the combination of visual processing, selective attention, and cognitive control. We therefore hypothesized that disruptions in the attentional control system may manifest in reduced activations in one or more of these components for SzP compared to HC during the visual search phase of the RSVP task.

Areas in the ventral attention network, on the other hand, are deactivated during the visual search phase until a target is detected and triggers a transient activation. Stronger deactivation of the ventral attention network during visual search is associated with improved selective attention as measured by improved correct detection of targets embedded in distractors (Shulman et al., 2007) or by a high- versus low-load working memory task (Todd and Marois, 2004). Therefore, we hypothesized that in ventral attention deactivation magnitude would correlate with detection rate in one or both populations, such that greater deactivation is associated with higher detection rate.

The balance of activation/deactivation of the dorsal and ventral attention network throughout the task requires intact connectivity both with the visual areas (which process the bottom-up inputs) and with the prefrontal cortex and the cingulo-opercular/salience networks, which together maintain cognitive control during the task (He et al., 2007). Therefore, we also examined the resting functional connectivity of these components. We hypothesized that there would be reduced connectivity between visual and other attentional control components and between components involving prefrontal cortex regions (cingulo-opercular/salience, dorsal attention) in SzP compared to HC. Further, we posited that connectivity between attentional control components would correlate with detection rate. Given the relationship between connectivity and functioning in the attentional control system, we also investigated the relationship between connectivity and functioning in predicting detection rate, hypothesizing that one might mediate the other.

## 2 Materials and methods

### 2.1 Participants

35 schizophrenia patients (SzP) and 34 healthy controls (HC), between ages 18–55 (Table 1), were recruited through the Lieber Schizophrenia Research Clinic (LSRC) with informed consent in accordance with New York State Psychiatric Institute’s Institutional Review Board (IRB). Participants completed a behavioral session and an MRI session, on separate days. The behavioral session included a demographic questionnaire as well as the Wide Range Achievement Test (WRAT-3) to measure IQ. During the behavioral session, SzP also completed the MCCB, a standard cognitive battery designed to test various domains of cognitive functioning in schizophrenia. All MCCB cognitive assessments were completed, with the exception of the behavioral tasks used to calculate the social cognition domain. In addition to the MCCB, SzP also received a 3rd behavioral session in which the Positive and Negative Symptom Scale (PANSS) was completed to determine severity of psychotic symptoms (Supplementary materials S1 for inclusion/exclusion criteria).

**Table 1:** Demographics, IQ, PANSS symptoms, and medications.

Demographics Mean (Standard Deviation)	Schizophrenia (n = 35)	Healthy controls (n = 34)	Statistics
Age (years)	39.4(11.2)	35.7 (9.8)	*t*(64) = 1.42, *p* = 0.16
Gender (male/female)	26/9	19/12	*χ^2^* = 1.28, *p* = 0.28
Race/Ethnicity (%White %Black **%Hispanic**)	31% 43% **34%**	26% 48% **13%**	*χ^2^* = 0.25, *p* = 0.61 *χ^2^* = 0.20, *p* = 0.65 ***χ^2^* = 4.1, *p* = 0.04**
Participant Education (years)	14.2 (2.7)	15.1(1.9)	*t*(61) = 1.5, *p* = 0.14
Participant SES	31.2 (13.4)	35.3(12.9)	*t*(64) = -1.3, *p* = 0.21
Edinburgh Handedness Score	17.5(4.2)	15.4(6.2)	*t*(52) = 1.6, *p* = 0.12
IQ (WRAT scaled score)	96.1(10.6)	99.4(13.6)	*t*(51) = -1.0*p* = 0.30
Parent SES	39.6(15.1)	43.2(13.9)	*t*(62) = -1.0, *p* = 0.32
PANSS Positive Symptoms	15.0(4.6)	–	
PANSS Negative Symptoms	13.5(4.1)	–	
PANSS Total Scores	56.6(14.1)	–	
Antipsychotic dose (CPZ equivalents, mg)	643.6(1070.6)	–	

### 2.2 Task fMRI and resting Acquisition, and processing

Two runs of the fMRI task and two to four resting state scans were collected on a 3 T GE Scanner for each participant. The fMRI task was adapted from a version of Patel and colleagues’ rapid-serial visual presentation (RSVP) paradigm (Patel et al., 2015, Patel et al., 2010), in which participants were instructed to fixate on a white dot appearing vertically below the center of the display screen, covertly attend to an RSVP stream of clip art images presented at 100 ms per image to the left, right, or center of the screen, and press a button when the target image appears (Fig. 1B and Supplementary Materials. Task duration was 5 min 30 s each (~20 trials/run). The event-related task design was designed to separate BOLD signals related to visual processing and attention from detection. Timing for correct detections, missed targets, and false positives, along with reaction times for correct detections were collected by the stimulus computer. Participants used a fiber optic button box for target detection. Eye movements were recorded using Eyelink 1000plus (SR Research, Mississauga, Ontario, Canada) to ensure participants were actively performing the task. Participants were briefly trained on the task, before BOLD collection began. At least two 5 min and 30 s resting state scans were collected before the RSVP task and two were collected after the completing the task.

Structural T1 and T2 (0.8 mm isotropic), multiband (MB) fMRI (2 mm isotropic, TR = 850 ms, MB factor 6), and distortion correction scans (B0 fieldmaps) were acquired as required for use of the Human Connectome Project (HCP) processing pipelines. The HCP pipelines performs standard preprocessing procedures (alignment to individual’s anatomical data, movement correction, distortion correction, and atlas alignment), along with surface-based extraction and surface atlas alignment of gray matter voxels to improve co-registration of functional maps between individuals and with standard surface atlases (Glasser et al., 2013). To ensure replicability, we aimed to collect ~20 min of resting state data per participant (Laumann et al., 2017).

### 2.3 Experimental design and statistical analyses

#### 2.3.1 Task fMRI, ROI Selection, and resting state analyses

BOLD runs in which participants were not performing the task or in which there were technical issues were excluded from the analysis, resulting in similar numbers of individuals in both groups with one and two task BOLD runs (32/34 HC with 2 BOLD runs, 35/35 SzP with 2 BOLD runs). Individual and group level task analyses were performed with FSL’s FEAT and FLAME toolboxes. The event-related design and analyses allowed for separation of sustained signals related to visual processing and attention from the transient signals related to detection (Fig. 1B) (Patel et al., 2015, Patel et al., 2010, Shulman et al., 2003). Analyses were first performed voxelwise to produce main effect activation surface maps for each group.

Regions of interest (ROIs) were drawn separately for each group based on activation maps, with labels largely based on sulcal/gyral anatomy for ROI-level comparisons of activation and connectivity (Supplementary Table 1; Supplementary Materials, S1.7.3 ROI Definitions). ROIs were then combined into *a priori* defined components (early visual, late visual, dorsal attention, ventral attention, cingulo-opercular/salience, prefrontal) to repeat analyses with reduced dimensions while preserving known functional/anatomical relationships. Prefrontal ROIs were separated from dorsal attention and cingulo-opercular components because they fell within the frontoparietal network at defined by Yeo et al., 2011, Gordon et al., 2014 and because of the separate status of prefrontal cortex in the original Corbetta-Shulman Model. Ventral attention ROIs were defined as voxels both deactivated by visual processing and attention and activated by detection; this was done in our earlier manuscript (Patel et al., 2015) and was similar to the original functional definition of the ventral attention network in Shulman et al. (2003). To avoid bias caused by separately defining ROIs within each group, analyses were repeated when ROIs were switched between groups (HC ROIs used in SzPs and vice-versa) and with ROIs derived from Gordon et al. (2014), with both control analyses yielding similar results (Supplemental Fig. 2). With the Gordon atlas, we also examined the activation of the angular gyrus node of the default mode network and its correlation with the detection rate.

Standard post-processing procedures adapted from Power et al. were used to minimize movement-related artifacts (Power et al., 2014), with a framewise displacement (FD) threshold of 0.2 mm for frame censoring (see Supplementary Methods and Results for further discussion). Resting state functional connectivity analyses were then performed by correlating the cleaned time-course of each ROI pair (Pearson’s correlations) within each participant. Between component connectivity was calculated by averaging all ROIs within a component, and then correlating the timecourses between components. Within-component connectivity was calculated by averaging ROI-ROI connectivity.

Spring-loaded graphs were produced in MATLAB and used to visualize the functional architecture of components and ROIs. The nodes in the graphs represent a single cortical component or ROI and were arranged such that the distance between any two nodes in the spring-loaded graph was inversely proportional to the similarity in their connectivity patterns with other areas. The connectivity strength between nodes was represented by the thickness of the line connecting the nodes, and the number and strength of connections to that node (weighted node degree) was represented by the diameter of the nodes. Weighted node degree was chosen as it provides information about which components are most critical for the functioning of a network (Betzel et al., 2016, Rubinov and Sporns, 2010).

#### 2.3.2 Linear modeling of relationship between Task, Connectivity, and behavioral measures

Pearson’s correlations were performed to examine the relationship between fMRI data and behavioral measures. To normalize the distributions, ROI-ROI or component-component resting-state connectivity data were Fisher-z transformed, and detection rate was arcsine-transformed. MCCB t-scores and ventral attention network deactivation were also used. 2 SzP were missing MCCB data due to attrition and were excluded from this analysis. All correlations across groups used group membership as a covariate. All variables were checked for correlation with antipsychotic medication dosage in chlorpromazine (CPZ) equivalents (Rose, 1997). If there was a significant correlation, the analysis was repeated with antipsychotic dosage as a covariate.

For multivariate relationships, stepwise linear regression was used to add or remove factors, with Bayes’ Information Criteria for penalized regression. Only resting state functional connectivity relationships prespecified by the modified Corbetta-Shulman model were considered for these analyses (Fig. 1).

#### 2.3.3 Multiple comparisons correction

Group differences in task activation data were corrected for multiple comparisons by Bonferroni correction. Due to the interconnectedness between ROIs inherent to resting state connectivity metrics, group differences in resting state connectivity and weighted node degree were corrected by non-parametric permutation testing (run 10,000 times) (Chen et al., 2016). All reported statistics survived multiple comparisons-correction unless otherwise noted.

See Supplementary Material for additional detailed information regarding task design, acquisition, and analysis procedures. All task and analysis materials will be made available to reasonable requests made to the corresponding author.

## 3 Results

### 3.1 Demographics and behavior

Demographics were closely matched between the two groups. SzP only significantly differed from HC group in percentage of individuals identifying as Hispanic (Table 1). Detection rate and false detection rate were similar in the two populations, but SzP reaction times were on average 46 ms longer than HCs (Table 2). Removing two outliers (>2sd) in the SzP reduces the mean RT to 577(56) milliseconds (ms), with a still-significant difference of 33 ms (p = 0.02). Reaction time strongly and negatively correlated with detection rate in both groups with no group difference in intercept or slope (reaction time: r_p_ = -0.45, F_2,70_ = 14.2, p = 0.0004). The proportion of individuals able to appropriately maintain fixation for at least 1 BOLD run was also similar between groups (24/35 SzP vs. 23/34 HC). In SzP, the range of MCCB domain scores ranged from a T-score of 40.31–42.56 (Supplementary Table 2) and detection rate correlated with the MCCB Attention/Vigilance t-scores (r = 0.42, p = 0.015), but not Speed of Processing, Working Memory, or Visual Learning. Detection rate did not correlate with antipsychotic dosage, as measured by CPZ equivalents (r = 0.25, p = 0.15).

**Table 2:** RSVP behavioral performance.

RSVP Task Performance Means (Standard Deviations)	Schizophrenia (n = 35)	Healthy controls (n = 34)	Statistics
Detection Rate (%)	75.1(19.5)	78.9(20.5)	*t*(67) = −0.8,*p* = 0.44
False detection rate (%)	60.1(61.4)	60.6(59.3)	*t*(67) = −0.03, *p* = 0.97
**Reaction Time (ms)**	**590(75)**	**544(59)**	***t*(65) = 2.84, *p* = 0.006**

### 3.2 Task fMRI results

**Fig. 2:**
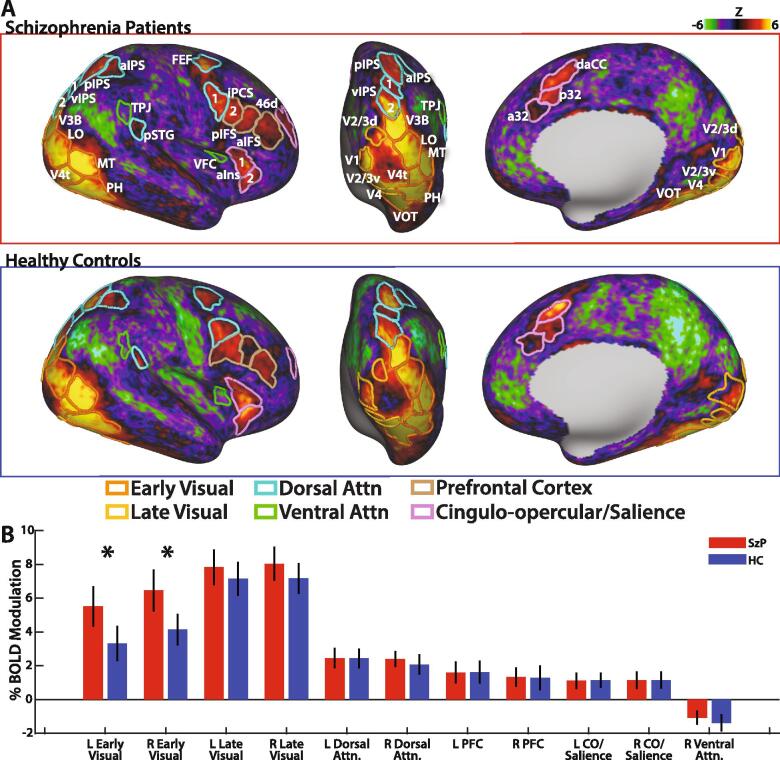
A) Group average mixed-effects activation maps represented on the cortical surface during the RSVP visual search phase. ROI definitions used for analyses are outlined in colors based on which component they were assigned to. B) Magnitude of activation for each component during the visual search phase of the task for SzP (red) and HC (blue). Error bars represent 95% confidence intervals, *p < 0.05 multiple comparisons corrected. Abbreviations: *L*: left *R:* right *CO:* cingulo-opercular *PFC:* prefrontal cortex *LO:* lateral occipital *MT:* medial temporal *VOT:* ventral occipital temporal *pSTG:* posterior superior temporal gyrus *TPJ:* temporoparietal junction *vIPS:* ventral intraparietal sulcus *pIPS:* posterior intraparietal sulcus *aIPS:* anterior intraparietal sulcus *FEF:* frontal eye-fields *iPCS:* inferior precentral sulcus *VFC:* ventral frontal cortex *aIns:* anterior insula *aMFG:* anterior middle frontal gyrus *aIFS:* anterior inferior frontal sulcus *pIFS:* posterior inferior frontal sulcus *a32:* anterior 32 *p32:* posterior 32 *daCC:* dorsal anterior cingulate cortex. (For interpretation of the references to colour in this figure legend, the reader is referred to the web version of this article.)

In both HC and SzP, ROIs defined by activation during the visual processing and attention or the visual search phase of the task were distributed through all four lobes, and were subdivided into early/late visual processing, dorsal attention network, prefrontal, and cingulo-opercular/salience components (Fig. 2A, Supplemental Table 1 and Supplemental Results). In addition, two ROIs defined by both deactivation during visual search and activation during detection were located in the TPJ and ventral frontal cortex (VFC) and assigned to the ventral attention network.

SzP showed significantly greater magnitude of activation in left (t_67_ = 2.8, p = 0.0062) and right (t_67_ = 3.0, p = 0.0037) early visual areas, compared to HC (Fig. 2B, both p < 0.05 multiple comparisons corrected). HC did not show greater activation in any components compared to SzP. Similar results were obtained when comparing the subpopulations of participants who were appropriately maintaining fixation.

### 3.3 Resting state functional connectivity results

**Fig. 3:**
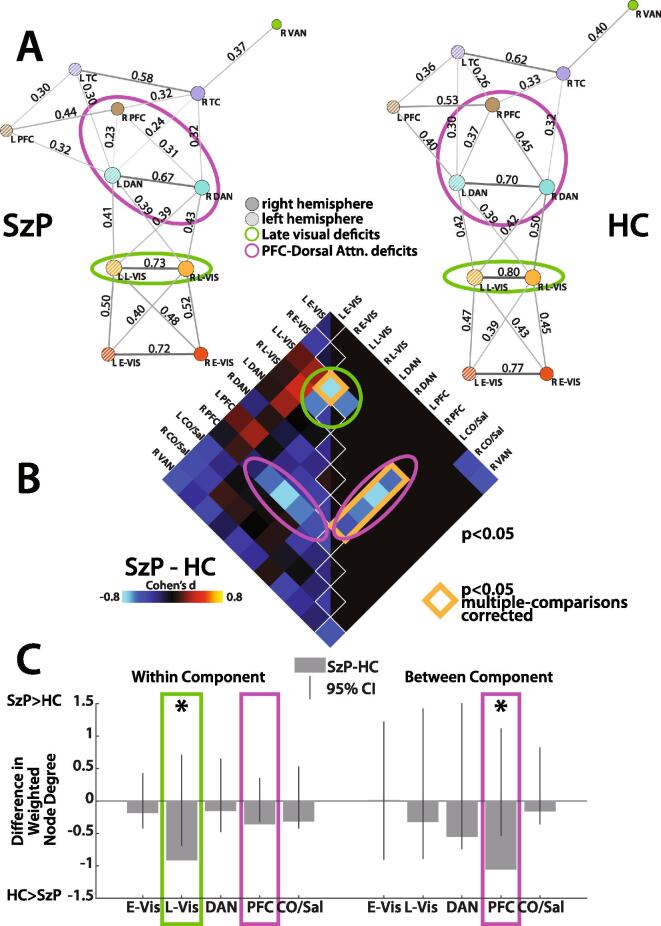
A) Spring-loaded network graphs of resting state functional connectivity based on weighted node-degree. Components thresholded at the 60th%tile, so that all nodes have at least one connection. Distance between nodes is inversely proportional to similarity in connectivity patterns, diameter of each node corresponds to number of connections, and thickness/darkness of edges corresponds to the strength of the connection. Component-component connectivity strength (r) noted next to each edge. Solid nodes represent right hemisphere components and striped nodes left hemisphere. Green outlines highlight late visual connectivity deficits and pink outline highlights prefrontal cortex deficits in all panels. B) Effect sizes of group difference in connectivity within and between components (11 × 11 components). Left half unthresholded, right half thresholded at p < 0.05. Orange outline highlights p < 0.05 multiple comparisons corrected differences, corrected through permutation testing. C) Group differences in within and between component connectivity, collapsed across hemispheres and represented as differences in weighted node degree (number of connections × strength of connections). Error bars reflect 95% confidence intervals (CIs) of the group differences derived from permutation testing. CIs may be asymmetric due to non-normal null distribution. *p < 0.05 multiple comparisons corrected. Abbreviations: *L*: left *R:* right *E-Vis:* early visual *L-Vis:* late visual *DAN:* dorsal attention network *PFC:* prefrontal cortex *CO/Sal:* cingulo-opercular/salience *VAN:* ventral attention network. (For interpretation of the references to colour in this figure legend, the reader is referred to the web version of this article.)

**Supplementary figure 1:**
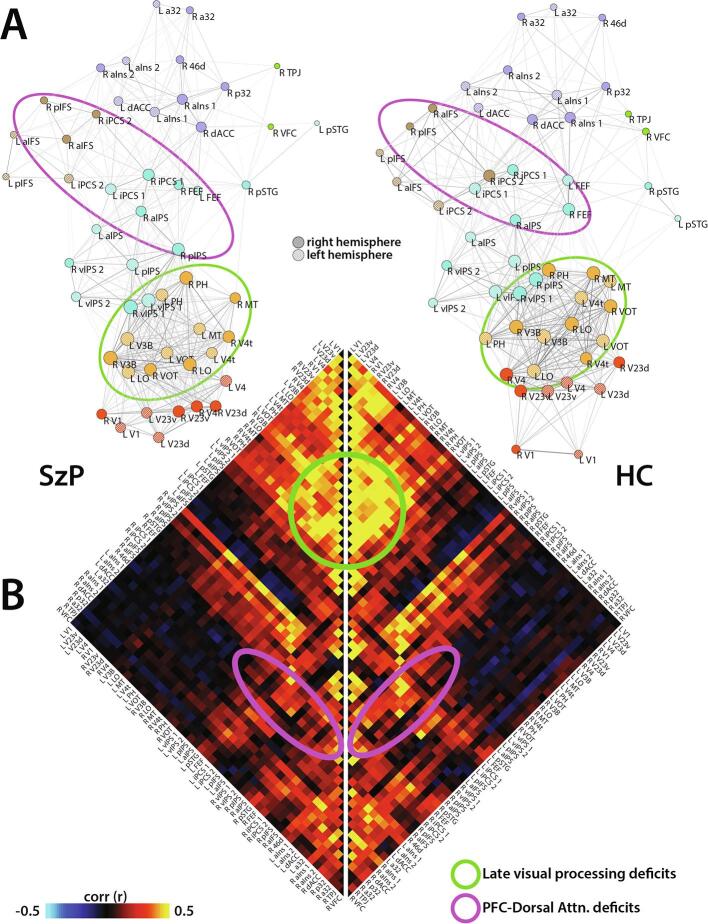


Spring-loaded graphs were used to visualize the resting state functional connectivity relationships in both groups (Fig. 3A). In HCs, the component graphs largely replicated the Corbetta-Shulman model, with visual components connected to cingulo-opercular/salience regions through dorsal attention and prefrontal areas (Fig. 3A versus Fig. 1). This includes the modification of the ventral attention network being connected to the cingulo-opercular/salience network. The hypothesized visual network to ventral attention network connection is missing in the component connectivity graph, but the ROI connectivity graph suggests that this may take place through a connection between the posterior superior temporal gyrus (pSTG) and the TPJ (Supplemental Fig. 1A).

While the overall functional architecture is qualitatively similar between groups (Fig. 3A), contrasting the component connectivity of HC and SzP reveals focal deficits both within and between components (Fig. 3B and Supplemental Figs. 1 and 2). We found the most pronounced within-component deficits in the connectivity of the left late visual cortex (d = 0.81, p = 0.0008. Comparing the within-component weighted node degree (the number and strength of connections between a node) after collapsing hemispheres confirmed this observation, with within-component connectivity deficits present in late visual cortex (Cohen’s d = 2.6, p < 0.05) along with a smaller deficit within prefrontal cortex that did not survive multiple-comparisons correction (Cohen’s d = 2.1, p < 0.05 uncorrected; Fig. 3C).

When examining between-component connectivity, we found deficits between right prefrontal cortex and multiple components, including left prefrontal cortex (d = 0.52), right dorsal attention (d = 0.61), left dorsal attention (d = 0.74), and right late visual (d = 0.58; Fig. 3B). The likelihood of observing deficits in the connectivity of the right prefrontal cortex and these four components by chance was p < 0.0001. Again, contrasting weighted node degree (after collapsing across hemispheres) confirmed our observation; between-component connectivity deficits were observed between prefrontal cortex and the other components (Cohen’s d = 2.5, p = 0.002; Fig. 3C). Results were similar when the connectivity analyses were repeated only for the subsets of HC and SzP who maintained fixation during the RSVP task (Supplemental Fig. 2). Antipsychotic dose in SzP did not correlate significantly with the strength of any component-component connectivity pairs.

### 3.4 Task deactivation of the ventral attention network and detection rate

**Fig. 4:**
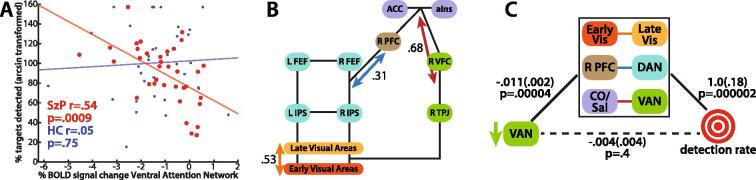
Visualizations of relationships between task activations, resting state functional connectivity, and detection rate A) Scatter plot showing that the correlation between ventral attention network deactivation and detection rate is significant for SzP (red) but not for HC (blue). B) Illustration of how detection rate and connectivity motif correlations map onto the Corbetta-Shulman Model illustrated in Fig. 1A. Orange double headed arrows represent detection rate with early to late visual components. Blue double headed arrows represent detection rate with dorsal attention to prefrontal components. Red double headed arrows represent ventral attention to cingulo-opercular/salience components. C) Illustration of mediation of ventral attention network and detection rate by 3 connectivity motifs. Unstandardized regression coefficients followed by standard error in parentheses. Green single headed arrow represents ventral attention deactivation. Abbreviations: *L*: left *R:* right *TPJ:* temporoparietal junction *IPS:* intraparietal sulcus *FEF:* frontal eye-fields *PFC:* prefrontal cortex *VFC:* ventral frontal cortex *aIns:* anterior insula *ACC:* anterior cingulate cortex *Early Vis:* early visual *Late-Vis:* late visual *DAN:* dorsal attention network *CO/Sal:* cingulo-opercular/salience *VAN:* ventral attention network. (For interpretation of the references to colour in this figure legend, the reader is referred to the web version of this article.)

Deactivation of the ventral attention network during visual search correlated strongly with detection rate in SzP, but not in HC (group × deactivation: F_2,67_ = 6.0, p = 0.017; SzP r = 0.54, p = 0.0009; HC: r = 0.05, p = 0.75; Fig. 4A). This relationship was similar in the subset of SzP and HCs who correctly maintained fixation (SzP: r = −0.46, p = 0.02; HC: r = 0.01, p = 0.95). Antipsychotic dose in SzP does not correlate with deactivation (r = 0.12, p = 0.50).

When examining the at the ROI level, the TPJ showed the strongest relationship with detection rate in SzP (r = -0.48, p = 0.0036; HC: r = 0.02, p = 0.90). Exploratory analyses revealed other activation-detection rate correlations within ROIs in the dorsal attention networks. However, there were no significant relationships between task activations with false detection rate or with reaction time (Supplemental Results). No correlation was observed between the deactivation of the adjacent angular gyrus node of the default mode network and detection rate (Supplemental Results). Similar results were also obtained when using HC ROIs to examine SzP data and vice-versa, suggesting that group-specific ROI definitions did not bias the results.

### 3.5 Predicting detection rate in SzP

We then used stepwise linear regression to examine the relationship between the strength of component-component connectivity and detection rate. Only three connectivity motifs, or pairwise correlations between two components, remained in the final model including the connectivity of right early to left late visual (partial r = 0.41, p = 0.005), the right dorsal attention to right prefrontal (partial r = 0.31, p = 0.02), and the left cingulo-opercular/salience to right ventral attention components (partial r = 0.39, p = 0.006; Fig. 4B). Together, the motifs explained 50% of the variance in detection rate (R^2^ = 0.50, p = 0.0001, F_2,33_ = 9.8).

When entered into a stepwise model for predicting detection rate with the three connectivity motifs, ventral attention network deactivation was eliminated, leaving the three connectivity motifs. Mediation analysis (Fig. 4C) revealed that the three connectivity motifs together mediated the relationship between ventral attention network and detection rate (Sobel test statistic = 3.6, p = 0.0003; (Mackinnon et al., 1995).

## 4 Discussion

In this study, we examined the activation patterns and resting state functional connectivity of components in the attentional control system in SzP and HC and their relationship to visual search performance. First, we found that performance on this task, as measured by detection rate, was similar between SzP and HC. Second, we found that early visual cortex was hyper-activated in SzP during the visual search phase of the RSVP task. Third, we found disruptions in the connectivity of late visual components and between prefrontal and other components (namely the dorsal attention network) in SzP. Fourth, we found that detection rate during the visual search task significantly correlated with deactivation of the ventral attention network in SzP, but not HC. Fifth, we found that detection rate correlated independently with connectivity between early to late visual, right prefrontal to dorsal attention, and cingulo-opercular/salience to ventral attention components of the attention control system. Lastly, we found that these three connectivity motifs mediated the relationship between detection rate and ventral attention deactivation during the visual search phase of the task. While the behavioral results may suggest a lack of attentional control deficits in SzP, the task and functional connectivity results point to a more intriguing picture: in the presence of late visual and prefrontal cortex deficits, some SzP may be able to use the attentional control system to compensate for these deficits.

### 4.1 Evidence of visual cortex anomalies

While the RSVP task was not designed to directly test visual processing integrity, the stimuli in this task involve a myriad of visual features that are processed in early and late visual areas, such as high contrast edges and implied motion. Subsequently, the findings of increased activation in early visual components and decreased functional connectivity within late visual cortex in SzP are consistent with previous behavioral, physiological, and fMRI studies in SzP. Behavioral studies have shown that compared to HC, SzP have reduced contrast sensitivity (Butler et al., 2005), impaired motion discrimination (Chen et al., 2004), and poor orientation discrimination (Tibber et al., 2015). Visual event-related potential studies have shown reduced amplitudes of P1 and N1 components to low contrast and low spatial frequency stimuli, indicating disruptions in the magnocellular visual pathway and possibly, dysfunction of NMDA-type glutamate receptors in SzP (Butler et al., 2007, Martinez et al., 2008). The visual connectivity deficits we see may therefore reflect a manifestation of the NMDA-receptor dysfunction of magnocellular inputs into these areas (Orliac et al., 2017, van de Ven et al., 2017). Increased activation in early visual components in SzP may also be the result of NMDA-receptor dysfunction in that Anderson *et al.* found increased receptive field width in areas V1-V4 may be linked to a NMDA-receptor hypofunctioning of inhibitory neurons (Anderson et al., 2016).

### 4.2 Significance of brain-behavior associations

While there was no relationship between ventral attention network activation with false detection rate, the correlation of the ventral attention network deactivation with detection rate in SzP may reflect a strategy to overcome visual processing anomalies by enhancing correct detection. The deactivation of the ventral attention network has long been thought to reflect the degree of engagement of the entire attentional control system (Shulman et al., 2007, Todd et al., 2005), and to our knowledge, this correlation is the first demonstration of this hypothesis. This increased use or possible compensatory strategy in SzP results in increased accuracy of detecting a target stimulus by both enhancing the processing of relevant stimuli and suppressing distractions by irrelevant stimuli (Corbetta et al., 2008). Conversely, the lack of brain-behavior correlations in HCs suggests that without visual processing limitations, a number of heterogeneous strategies may be employed for accurately detecting the target stimuli.

The deficits in the connectivity between prefrontal cortex and dorsal attention network and the correlation between detection rate with this motif points to the importance of the communication between these two components in the control of attention. The prefrontal areas activated in this task are part of the frontoparietal network (Yeo et al., 2011) that are implicated in communicating with multiple brain regions to maintain and update current task demands (Dosenbach et al., 2008). Disruptions in these lateral prefrontal areas have been linked to working memory and cognitive control processes including deficits in goal maintenance in SzP (Poppe et al., 2016). In contrast, the dorsal attention areas (specifically pIPS and FEF) are retinotopically organized maps that represent attentional priority—i.e., where in space one’s attention is currently being allocated (Bisley and Goldberg, 2010, Jerde and Curtis, 2013). Failures in the communication between the two components may then lead to or reflect the failure to allocate enough priority and or attention to a spatial location: in this case, the location of the RSVP stream. The combination of the deficit and the correlation in these two components may suggest these connections are not only critical to the control of attention, but that some SzP have more severe deficits in these connections that prevent them from effectively using the entire attentional control system as a part of a compensatory strategy.

The other connectivity motifs that correlated with performance—cingulo-opercular/salience to ventral attention and early to late visual components—were not disrupted in SzP. Therefore, these correlations may reflect increased use of (or the ability to use) the attention control system to overcome the visual processing deficits. The increased cingulo-opercular/salience to ventral attention connectivity may reflect greater use of the ventral attention network to avoid distractions, whereas the increased early to late visual cortex connectivity may reflect increased frequency and intensity of top-down signals from the dorsal attention network to enhance visual representations shared between early and late visual areas (Corbetta et al., 2008, Squire et al., 2013).

The observation that connectivity of all three major connectivity motifs—early to late visual cortex, cingulo-opercular/salience to ventral attention, and prefrontal to dorsal attention—contribute independent variance in detection rate indicates either that there are multiple potential deficits in SzP affecting visual attention/processing and/or that full compensation requires the intact communication of major components of the Corbetta-Shulman model. The mediation of the ventral attention network deactivation and detection rate by the connectivity motifs supports this, suggesting that the intact system is necessary for the maximal engagement of the attentional control system (Corbetta et al., 2008). The fact that these interactions were predicted by the model highlights its explanatory power in describing the ROI and component interactions implicated in attentional control.

### 4.3 Limitations

There are a number of caveats to these findings, namely that the chosen task was not designed to interrogate specific functions of any given component or ROI involved in the attentional control system. Results from seemingly similar studies may also differ from ours for a number of reasons, including a) whether the stimuli evoked the specific visual processing deficits observed in SzP (Anderson et al., 2016, Martinez et al., 2012b, Orliac et al., 2017, van de Ven et al., 2017), b) whether the task chosen loads more onto prefrontal-based cognitive control processes through frontoparietal or cingulo-opercular/salience networks as opposed to selective attention processes controlled by dorsal/ventral attention networks (Jimenez et al., 2016, Wynn et al., 2015) (see Supplementary Discussion), and c) whether the imaging/parcellation methods rely on coarser resting-state parcellation schemes or volume-based coordinates that result in mixing of signals between adjacent areas (Glasser et al., 2016). Another potential limitation was the high average antipsychotic medication dose in our SzP population.

### 4.4 Future directions

This study provides a framework to guide future investigations of the mechanisms of and the potential treatment for visual processing and attention deficits in SzP. Testing the inferences made about the deficits and correlations observed in this study with different tasks and with causal manipulations (such as TMS) will be crucial to disentangling deficits from compensatory mechanisms. In particular, further study of the integrity of the dorsal and ventral attention networks is needed, such as the quantification of receptive field structure (Mackey et al., 2017), which may reflect microcircuit deficits not easily seen with the present task (Murray et al., 2014). Connections between early to late visual cortex, right dorsal attention to right prefrontal, or left cingulo-opercular/salience to right ventral attention may be promising targets for neuromodulation either to improve or suppress attentional control functioning to force use of the visual networks—similar to what is being investigated in stroke rehabilitation (Corbetta, 2014). The use of the attentional control system to overcome visual processing deficits may also point to a compensatory mechanism that may generalize to any disorder with visual deficits. Lastly, these results demonstrate the utility of close application of cognitive neuroscience models to the study of psychiatric disorders.

## CRediT authorship contribution statement

**Sophie C. Arkin:** Writing - original draft, Writing - review & editing, Investigation, Formal analysis, Visualization. **Daniel Ruiz-Betancourt:** Formal analysis, Software, Visualization, Writing - original draft. **Emery C. Jamerson:** Investigation, Methodology, Software. **Roland T. Smith:** . **Nicole E. Strauss:** Investigation. **Casimir C. Klim:** . **Daniel C. Javitt:** Funding acquisition, Resources. **Gaurav H. Patel:** Conceptualization, Methodology, Formal analysis, Funding acquisition, Resources, Software, Writing - original draft, Writing - review & editing, Supervision.

## Declaration of Competing Interest

GHP receives income and equity from Pfizer, Inc through family; DCJ has equity interest in Glytech, AASI, and NeuroRx. He serves on the board of Promentis. He holds intellectual property rights for the use of NMDAR agonists in the treatment of schizophrenia, NMDAR antagonists in the treatment of depression and PTSD, and has submitted disclosures for fMRI-based prediction of ECT and TMS response, and EEG-based diagnosis of neuropsychiatric disorders. Within the past 2 years, he has received consulting payments/honoraria from Cadence, Biogen, SK Life Science, Autifony, Glytech and Boehringer Ingelheim.  SCA, DRB, ECJ, RTS, NES, and CCK reported no biomedical financial interests or potential conflicts of interest.

## Figures and Tables

**Supplementary figure 2:**