# Supplementary material for: Deficits and compensation: Attentional control cortical networks in schizophrenia
Source: Neuroimage Clin. 2020 Jul 20;27:102348. doi: 10.1016/j.nicl.2020.102348 (PMC7393326; doi:10.1016/j.nicl.2020.102348)
Supplement: Supplementary data 1 [file mmc1.docx]

**S1. Supplementary Methods and Materials**

*S1.1 Inclusion/ Exclusion Criteria*

Participants were primary English speakers, devoid of history of head trauma and neurological disorders, and had normal or corrected visual acuity (20/40 or better). Participants with contraindication for MRI (artificial implants and/or claustrophobia) were excluded from the study. Patients met DSM-IV criteria for schizophrenia, as confirmed by the Lieber Schizophrenia Research Clinic (LSRC) before recruitment and participation. Schizophrenia patients (SzP) were on disease-appropriate medication used within appropriate guidelines and were assessed with the Positive and Negative Symptoms Scale (PANSS; (Kay et al., 1987). Patients scoring above 120 on the PANSS, or those who were actively psychotic were excluded. Healthy controls (HC) were recruited through the LSRC and through IRB-approved flyers and internet advertisements. The Structured Clinical Interview for the DSM-NP (SCID-NP) was used to exclude past or present Axis I or II disorders, significant substance use disorders in the past 6 months, and in HC significant psychiatric family history (First et al., 1997).

*S1.2 Apparatus and Procedural Setup for fMRI*

Participants were placed on the scanner bed with cushioning to stabilize potential head movement and earplugs to block out MR-related sound. A mirror attached to the head coil allowed participants to view visual stimuli displayed on a translucent screen mounted at the end of the bore from a liquid crystal display (LCD) projector; the eye-to-screen distance (mirror-to-screen + eye-to-mirror) was 107 cm. Participants held a fiber optic, five-channel button box (Current Designs, Philadelphia, PA) in their right hands that allowed them to respond to target stimuli with their index finger. The experimental display was controlled by a MacBook Pro (Apple Inc., Cupertino, CA) running custom software in MATLAB (Mathworks, Natick, MA) using Psychophysics toolbox version 3.012 (Brainard, 1997; Pelli, 1997), which connected to the projector via high-definition multimedia interface (HDMI) hub. The stimuli were synchronized to the beginning of the MR volume acquisition via a USB device that triggered the MATLAB paradigm to begin (Current Designs, Philadelphia, PA).

*S1.3 fMRI Acquisition*

Functional and anatomical data were acquired with a 32-channel phased array receive-only head coil (Nova Medical, Wilmington, MA) by General Electric’s Discovery MR750 3.0 Telsa full body MR scanner (GE; Fairfield, CT) at New York Psychiatric Institutes’ (NYSPI) MRI Research Unit. Localizer scans and a gradient echo B0 fieldmap (TE=4.8ms, TR=800ms, FOV=256mmx256mm, slice thickness=2mm, matrix=128x128, slices=68) were required for the Human Connectome Project (HCP) processing pipelines. Structural images were acquired in 5-minute sequences and were comprised of two T1-weighted images (3D sagittal, 0.8mm isotropic, matrix size=300x300, slices=220, TR=7856ms, TE=3108ms, flip angle=12°, TI=450ms) and two T2- weighted images (3D sagittal, 0.8 mm isotropic, matrix size=320x320, slices=220, TR=2500ms, TE=95.708, flip angle=90°). Task and resting state functional data were collected with a multiband SMS-EPI sequence (2 mm isotopic, slice plane=transverse, TR=850ms, MUX=6, ARC=1, TE=25ms, matrix size=96x96, slices=66, phase encoding direction=P🡪A) (courtesy of the Center for Cognitive and Neurobiological Imaging, Stanford University, http://cni.stanford.edu).

*S1.4 RSVP fMRI Paradigm*

Forty-two clipart images of neutral objects were used as both target and distractor stimuli. One of the forty-two clipart images was randomly selected as the target image, while the other forty-one images were used as distractors. Before the BOLD run started, the target image was presented once in each of the three stream locations to familiarize the subject with the target. One stream was superimposed over the fixation point in the center of the screen with a width/height of 3.6°. The two peripheral streams were located at 5.8° eccentricity with a polar angle of 0° (left of the fixation point) and 180°(right of the fixation point) and with a width/height of 5.3°. Targets were chosen randomly, under the condition that no target could be used twice in a row. Participants pressed a specified button on a button box with their right index finger every time they saw the target stimulus.

Each trial consisted of a 10 second RSVP stream in one of three locations and with the same sizes described above. Each stimulus in the RSVP stream was displayed for 100 milliseconds (ms) before being replaced by the next distractor with no gap between presentations. Distractor stimuli were chosen randomly with the constraint of not having consecutive repeats. The target stimulus took the place of a distractor stimulus in the stream and did not differ in any parameters from the distracting stimuli. Half of all trials had a single target presented randomly within the first 8 seconds in the 10-second stream. One-quarter of trials had no target (catch trials). In the remaining one-quarter of trials, the target appeared twice within the target stream, once within the first 4 seconds and again from 4 to 8 seconds. After the target appeared, participants had 1.3 seconds to indicate detection. Participants were instructed to respond as accurately and quickly as possible, with an emphasis on accuracy so as to minimize false positives. Following each 10-second stream, the participant was asked to maintain fixation for inter-trial intervals of 2, 4, or 6 seconds, chosen randomly, before the next trial began.

*S1.5 Resting State Acquisition*

For the resting state scans, participants were asked to fixate on a white dot (.3°x.3°) in the center of a black screen for 5 m 30 s runs. At least two resting state scans were collected before the RSVP task and two were collected afterward. Resting state scans where participants fell asleep were documented during data collection and were excluded from all analyses. The number of resting state scans per subject did not differ between group (p=.28), with 27/34 HCs and 26/35 SzPs with 4 or more BOLD runs of resting state data.

*S1.6 fMRI Processing*

All imaging data were processed on workstation machines (Mac Pro, Apple Inc., Cupertino, CA) using The HCP processing pipeline v3.4 adapted for the NYSPI GE MR750 MRI scanner. The HCP pipelines first processed the anatomy images to create a cortical surface model for each individual aligned to the HCP fs_LR 32k atlas, then for the functional runs it performed movement correction, distortion correction, and atlas alignment in a single resampling step, and lastly projected the functional data to an atlas cortical surface through the individual’s cortical surface model (Glasser et al., 2013). After each step, manual quality checks were performed to check for errors in atlas-alignment and segmentation. This pipeline is different from other processing pipelines in that it creates a Connectivity Informatics Technology Initiative (CIFTI) file for each BOLD run that only contains the data from the cortical and the subcortical grey matter (“gray-ordinates” as opposed to voxels), which allows for a more precise localization of brain activity that is not confounded by cerebrospinal fluid (CSF) or white matter partial volume effects. Structural and functional images were aligned in a volume space of the Montreal Neurological Institute (MNI152) atlas and on the surface Conte69/fs_LR 32k atlas created by the HCP pipeline developers (Evans et al., 1992; Glasser et al., 2013; van Essen et al., 2012).

*S1.7 Experimental Design and Statistical Analyses*

*S1.7.1 Task fMRI Analyses*

For level 1 analyses, the magnitudes of activation evoked by attention to and processing of the RSVP stream and detection of the target at each spatial location were separately estimated in each individual via a general linear model (GLM) by modeling each event type as an independent regressor (Shulman et al., 2003). For the RSVP stream, the regressor was formed by convolving a 10 second boxcar with an estimate of the hemodynamic response to an impulse function (Grinband et al., 2017). For detection events, the reaction time of the response to each target was used as the boxcar that was convolved with the impulse response. For the misses and false detections, the event duration used was 500ms. A high-pass filter was of .0031 Hz (1/325s) was applied to the BOLD data and the regressors. The first four volumes of each run were excluded from the analyses. Results from individuals with two BOLD runs were combined in a level 2 fixed-effects analysis. Level 3 group analyses were performed using FLAME 1 mixed-effects.

With 25% of trials not containing any targets (catch trials), this design allowed for adequate separation of the RSVP stream and target detection events, similar to the catch trial design validated in (Ollinger et al., 2001b) and (Ollinger et al., 2001a) and used in {Patel:2010p5720}. For this experiment, the design matrices were invertible with an average matrix condition number of 9.3 and an average correlation between the RSVP stream and target detection regressors of 0.49 across all runs and participants.

*S1.7.2 Lateral Preference Analyses*

Eye-traces were manually inspected to determine whether each subject maintained fixation within a 1° window around the fixation point or was making saccades to the stream location when presented in the periphery. 23 HCs and 24 SzPs had one or both BOLD runs in which they maintained fixation (16/23 HCs with 2 BOLD runs, 21/24 SzP with 2 BOLD runs). The task analyses were repeated in only these subjects, and in addition activation for contralateral vs. ipsilateral RSVP streams were used to calculate an index and compare the groups (Patel et al., 2010).

*S1.7.3 ROI Definitions*

For each group, regions of interest (ROIs) for the low/high-level visual processing, dorsal attention, and prefrontal regions were defined on mixed-effects average activation maps on the cortical surface by drawing borders around each activation. Activation gradient maps derived from the spatial derivative of the z-statistic maps were used to define borders around local maxima (peaks), and to separate contiguous activations by tracing the line that represented the highest gradient (local minimum) between the two activations (Glasser et al., 2016). ROI labels were largely based on sulcal/gyral anatomy, with the HCP’s multi-modal parcellation of the cortical surface serving as a guide for labeling some of the ROIs. Foci spanning several HCP ROIs were labeled according to the main overlapping ROI (Glasser et al., 2016). Ventral attention ROIs (right hemisphere TPJ and VFC) were drawn for each group on the mixed-effects maps of the conjunction of gray-ordinates a) deactivated by the RSVP monitoring and b) activated by target detection (Patel et al., 2015). The TPJ was limited to the posterior bank of the supramarginal gyrus, and the VFC to insular cortex posterior to the anterior insula ROIs.

Component label assignments were based on anatomical location in relation to previous studies. Group-average ROIs drawn in the HCP fs_LR 32k surface atlas were projected to each individual’s fs_LR 32k-registered cortical surface and used to extract BOLD activity evoked by RSVP monitoring and target detection at each stream location and for the sum of the three stream locations (Glasser et al., 2013). Validation of ROI-level results was performed using the Gordon *et al.* parcellation scheme (Gordon et al., 2014), by selecting ROIs significantly covered by that task activations (see **Supplementary Table 1**).

*S1.7.4 Resting State Functional Connectivity Analyses*

Additional post-processing procedures, adapted from Power et al. (Power et al., 2014), were performed to minimize artifact in the resting state functional connectivity analyses. First, estimates of head motion calculated in the X, Y, and Z directions along with the displacements of rotation around the X, Y, and Z axes (pitch, yaw, and roll) by the HCP movement correction algorithm, along with their derivatives (backwards difference), the square of the six parameters, and the sum of these six parameters (assuming a 50mm radius of head size for the rotations, previously labeled as the framewise displacement (FD)) were used as nuisance regressors to remove movement-related artifact. Second, tissue signals and their derivatives calculated by averaging signal across voxels within a spatial mask for ventricle signals, CSF signal, white matter signal, and whole brain signal were used as nuisance regressors to remove physiology-related noise (global signal regression also removes movement-related artifact (Power, 2016). Third, MR frames with an FD>0.2mm were censored, and replaced by interpolation using a method based on the Lomb-Scargle periodogram (Lomb, 1976; Power et al., 2014). Fourth, data were low band-pass filtered at .588 Hz (the Nyquist limit for the MR sampling rate) and high band-pass filtered at 0.0005 Hz (Glasser et al., 2013). The resulting time-series was used for subsequent resting state functional connectivity analyses.

The cleaned time-courses were used to calculate the connectivity between each pair of ROIs by first parcellating the time-courses for each ROI and then calculating the correlation between the time-courses for each ROI pair (Pearson correlation). Component-Component connectivity was again calculated in two ways: 1) averaging the Fisher-z transformed connectivity for each ROI-ROI interaction in each system pair (ROI averaging), and 2) by combining the component ROIs into a single parcel, parcellating the voxelwise time series, and then calculating the connectivity between each pair of systems (time-course averaging). Censored frames were omitted from all correlation calculations. For group average connectivity matrices, each individual’s connectivity matrix was Fisher-z transformed, averaged, and then the inverse Fisher-z was applied to the result. Group comparisons were performed using two-sample t-tests on the Fisher-z transformed individual data. Spring-loaded graphs were created by thresholding the ROI-ROI correlation matrix at the lowest percentile for each group in which each node possessed at least 2 connections, which was the 65^th^%tile for the ROI graphs and the 60^th^%tile for the component graphs. Using the Pajek software suite (http://pajek.imfm.si/), the weighted node-degree for each node was calculated, and then nodes were distributed using the Kamada-Kawai free-energy separate components option. Within/between component connectivity scores were calculated by the average connectivity strength of component ROIs with other ROIs in the same or other components.

*S1.7.5*

For group comparisons, group labels were shuffled between participants 10,000 times, and on each shuffle the Cohen’s d was calculated for each component-component connectivity strength. For within component connectivity, the p-value reflects the likelihood of a difference with that effect size. For between-component connectivity, the Cohen’s d difference map was first thresholded at p<0.05. Then, the likelihood of the remaining components passing that threshold together by chance was calculated for the p-value.

Group differences in node degree were also corrected by permutation testing. Group labels were again shuffled between participants 10,000 times, and on each shuffle the average within and between component node degree was calculated. The p-value represents the likelihood of obtaining an effect size difference of the magnitude found in the actual data.

**S2. Supplemental Results**

*S2.1 Topography of Task Activation*

Visual areas included striate/extrastriate visual areas (yellow boundaries in **Figure 2A**) and higher-level object recognition areas in lateral/ventral occipital cortex (orange boundaries). Labels for these areas were largely derived from the HCP atlas (Glasser et al., 2016); some of the activation foci spanned multiple HCP areas and were given a single label from the largest or most recognizable area within the set. Dorsal attention areas included a series of areas ascending along the intraparietal sulcus (IPS) that corresponded to previously identified parietal nodes of the dorsal attention network (Corbetta et al., 2008; Patel et al., 2015), and overlap with previously identified retinotopically organized parietal areas (Jerde and Curtis, 2013; Silver and Kastner, 2009). The foci loosely resembled the three foci described by Corbetta and colleagues (Corbetta et al., 2008), with ventral IPS (vIPS) subdivided into 2 foci overlapping with area V7 (vIPS 1) and IPS 1 (vIPS 2) in the HCP parcellation; posterior IPS (pIPS) overlapping with HCP parcels LIPv/d and roughly IPS 2/3/4 in Silver and Kastner 2009 and IPS 2/3 in Jerde *et al.* 2013; and aIPS with LIPv/posterior AIP in the HCP atlas and IPS 5 in Silver and Kastner 2009 (Jerde and Curtis, 2013; Silver and Kastner, 2009). Prefrontal dorsal attention network activations spanned the precentral sulcus. These were subdivided into the frontal eye-fields (FEF) in the HCP parcellation (sPCS in Jerde *et al.*) and inferior precentral sulcus (iPCS 1;PEF in the HCP parcellation). Prefrontal cortex areas extended anteriorly along the inferior frontal sulcus and overlapped with a number of HCP parcels: iPCS 2 (iFJp and 6r), pIFS (IFJa), and aIFS (IFSp and 9/46v) (see legends for **Figure 2** and **Supplemental Figures 1** and **2** for abbreviation definitions). These areas were contained within the frontoparietal network as defined by Yeo *et al.* (Yeo et al., 2011) and Gordon *et al.* (Gordon et al., 2014). We observed an additional activation on the posterior superior temporal gyrus (pSTG) that is not part of the Corbetta-Shulman dorsal attention network, but likely corresponds to a visually responsive area labeled ventral TPJ (vTPJ) by Horiguchi *et al.* in both groups (Horiguchi et al., 2016). Activations in lateral prefrontal cingulo-opercular/salience areas were located on the anterior pole (46d, overlapping HCP 9/46d) and in the anterior insula (aIns, subdivided into two foci and overlapping HCP FOP4, FOP5, and AVI). In medial prefrontal cortex, activated cingulo-opercular areas included dorsal anterior cingulate cortex (dACC, overlapping parts of HCP SCEF and 8BM) and anterior area 32 (a32, overlapping HCP a32pr), with an additional right hemisphere-only activation in posterior area 32 (p32, overlapping HCP p32pr). These areas overlapped with both the cingulo-opercular and salience networks as defined by Yeo *et al.* (Yeo et al., 2011) and Gordon *et al.* (Gordon et al., 2014).

The two right hemisphere ventral attention areas were defined by the conjunction of areas *deactivated* by the RSVP stream and *activated* by detection, similar to the functional definitions used to define ventral attention areas in Shulman *et al.* (Shulman et al., 2003) and Patel *et al.* (Patel et al., 2015). The TPJ matched the R Cingulo-opercular 29 parcel in Gordon *et al.* (Gordon et al., 2014), and the VFC matched FOP3 in HCP and R Cingulo-opercular 33 in Gordon *et al.*

*S2.2 ROI Activation Correlations with Detection Rate*

In addition to TPJ deactivation, weaker detection-rate correlations at the ROI level included the L and R ventral intraparietal sulcus (vIPS) 1 (r=0.41, p=0.01; r=0.31, p=0.04) and R inferior precentral sulcus (iPCS) 1 (r=0.38, p=0.02) for SzP. In HCs, L V1 demonstrated a potential relationship with detection rate (r=-0.43, p=0.01).

*S2.3 Ventral Attention versus Default Mode Network*

Hyperactivity (or reduced deactivation) of the default mode network has sometimes been implicated in reduced control of selective attention (Whitfield-Gabrieli and Ford, 2012). In addition, one study questioned whether the right temporoparietal junction was actually separate from the right angular gyrus node of the default mode network (Kubit and Jack, 2013). In our results, the right angular gyrus is strongly deactivated in both groups (Figure 2A). We therefore conducted post-hoc analyses to examine whether the deactivation of the right angular gyrus and correlated with detection rate, as was seen in the right TPJ.

Since our localizers were not designed to define areas within the default mode network, we used the Gordon atlas (Gordon et al., 2014) to define the ROIs: R Default 26 for the right angular gyrus and R Cingulo-Opercular 29 for the right TPJ. The right angular gyrus was similarly deactivated in both groups (t_67_=-0.64, p=0.52). However, there was no significant correlation with detection rate across the two groups (F_2,67_=0.31, p=0.58). There also was not any group x deactivation interaction (F_2,67_=0.22, p=0.64) with no correlation in either the SzP (r=-0.14, p=0.4) or the HC (r=-0.02, p=0.9).

For the right TPJ, deactivation again was similar in the two groups (t_67_=-1.8, p=0.07). The main effect of group was not signifcant (F_2,67_=2.9, p=0.10) but there was a significant group x deactivation interaction (F_2,67_=4.19, p=0.045), with a significant correlation in the SzP (r=-0.50, p=0.0025) but not in HC (r=0.01, p=0.95). Of note, the right TPJ results here confirm those reported in the main manuscript with an independently defined ROI.

*S2.4 Contralateral versus Ipsilateral Activation*

In the subset of subjects who maintained fixation, there was no difference in the amount of activity evoked by a contralaterally vs. ipsilaterally located RSVP stream in any ROI.

*S2.5 Resting State Frame Censoring*

Overall, SzP had more frames censored due to motion: 732.1 frames for SzP (10.4 minutes, 44% of total frames) versus 471.3 frames for HC (6.7 minutes, 31% of total frames). However, more frames of resting state data were collected in SzP high motion participants, resulting in a similar number of frames remaining for analysis: 882.9 frames for SzP (12.5 minutes) versus 1078.1 frames for HC (15.3 minutes) (t_67_=2.1, p=0.04). All participants except 2 HC had more than 5 minutes of resting data after censoring.

**S3. Supplemental Discussion**

*S3.1 Previous Findings*

Two recent studies have found deficits in the activation/deactivation of both dorsal and ventral attention networks, along with behavioral deficits in the tasks used to evoked these differences (Jimenez et al., 2016; Wynn et al., 2015). Our results complement theirs, providing further insights into the relative function of attentional control and visual processing in schizophrenia. Whereas our task largely relies on feature-based visual discrimination of novel pictures of objects, operations that largely take place in the interactions of the dorsal/ventral attention networks and visual cortex (Corbetta et al., 2008; Squire et al., 2013), the two previous studies’ tasks relied on discrimination of over-learned sparse stimuli (letters and numbers) that either switched identity as target or distractor between sessions (Wynn et al., 2015) or involved memory of previous stimuli (dual-task trials in (Jimenez et al., 2016)). These tasks likely rely more on cognitive-control networks to de-conflict otherwise prepotent responses (Shenhav et al., 2016), resulting in differential activation patterns stemming from different task demands. The experiment analogous to ours from these two studies are the single-target trials in Jimenez *et. al.* (2016); despite not being able to separate target detection from visual processing/attention signals, they observe some compatible effects, including greater activation of the dACC (similar to the increased activation of the cingulo-opercular/salience network we see for target detection) and deactivation of the TPJ in SzP (potentially from SzP maximally deactivating the TPJ in an attempt to overcome a task-control deficit). In addition to the visual cortex deficits we observed, these results may point to a deficit in cognitive control areas, which have previously been reported in resting state functional connectivity studies (Baker et al., 2013) and in fMRI studies involving working memory (Van Snellenberg, 2009). Therefore, depending on the task as well as task-demands, one or both deficits may contribute to the overall appearance of attention deficit in SzP.

**Supplementary Table and Figure Captions:**

**Supplementary Table 1:** This table shows how the RSVP functionally defined ROIs map onto previously published parcellation schemes and studies.

**Supplementary Table 2:** This table shows the average T-score for each cognitive domain of the MATRICS Cognitive Consensus Battery (MCCB) with the exception of the social cognition domain.

**Supplementary Figure 1:** SzP (right) and HC (left) ROI-ROI resting state functional connectivity results **A)** Spring-loaded graphs of ROI-ROI resting state connectivity. Distance between nodes is inversely proportional to similarity in connectivity patterns, diameter of each node corresponds to number of connections, and thickness/darkness of edges corresponds to strength of connection. Darker nodes correspond to ROIs found in right hemisphere and lighter nodes to left hemisphere. **B)** ROI-ROI connectivity matrices (52 x 52) . Green outlines highlight late visual processing deficits and pink outline highlights prefrontal to dorsal attention component deficits. Abbreviations: *L*: left *R:* right *PFC:* prefrontal cortex *LO:* lateral occipital *MT:* medial temporal *VOT:* ventral occipital temporal *pSTG:* posterior superior temporal gyrus *TPJ:* temporoparietal junction *vIPS:* ventral intraparietal sulcus *pIPS:* posterior intraparietal sulcus *aIPS:* anterior intraparietal sulcus *FEF:* frontal eye-fields *iPCS:* inferior precentral sulcus *VFC:* ventral frontal cortex *aIns:* anterior insula *aMFG:* anterior middle frontal gyrus *aIFS:* anterior inferior frontal sulcus *pIFS:* posterior inferior frontal sulcus *a32:* anterior 32 *p32:* posterior 32 *daCC:* dorsal anterior cingulate cortex

**Supplementary Figure 2**: Control Analyses **A)** Connectivity contrast matrices using groups’ own ROIs (left) vs. reversing group ROIs (right). **B)** Connectivity contrast matrices using groups’ own ROIs (left) vs. an independently defined parcellation scheme (Gordon *et al.* 2014). **C)** Connectivity contrast matrices for full dataset (left) vs. the subset of HC (n=16) and SzP (n=21) who maintained fixation throughout the two runs of data collection. Green outlines highlight late visual processing deficits and pink outline highlights prefrontal to dorsal attention component deficits. Abbreviations: *L*: left *R:* right *PFC:* prefrontal cortex *LO:* lateral occipital *MT:* medial temporal *VOT:* ventral occipital temporal *pSTG:* posterior superior temporal gyrus *TPJ:* temporoparietal junction *vIPS:* ventral intraparietal sulcus *pIPS:* posterior intraparietal sulcus *aIPS:* anterior intraparietal sulcus *FEF:* frontal eye-fields *iPCS:* inferior precentral sulcus *VFC:* ventral frontal cortex *aIns:* anterior insula *aMFG:* anterior middle frontal gyrus *aIFS:* anterior inferior frontal sulcus *pIFS:* posterior inferior frontal sulcus *a32:* anterior 32 *p32:* posterior 32 *daCC:* dorsal anterior cingulate cortex

**Supplemental Materials References:**

Baker, J.T., Holmes, A.J., Masters, G.A., Yeo, B.T.T., Krienen, F., Buckner, R.L., Ongür, D., 2013. Disruption of Cortical Association Networks in Schizophrenia and Psychotic Bipolar Disorder. JAMA Psychiatry. doi:10.1001/jamapsychiatry.2013.3469

Brainard, D.H., 1997. The psychophysics toolbox. Spatial Vision.

Corbetta, M., Patel, G.H., Shulman, G.L., 2008. The reorienting system of the human brain: from environment to theory of mind. Neuron 58, 306–324. doi:10.1016/j.neuron.2008.04.017

Evans, A.C., Marrett, S., Neelin, P., Collins, L., Worsley, K., 1992. Anatomical mapping of functional activation in stereotactic coordinate space - ScienceDirect.

First, M.B., Spitzer, R.L., Gibbon, M., Williams, J.B.W., 1997. Structured Clinical Interview for DSM-IV® Axis I Disorders (SCID-I), Clinician Version, Administration Booklet. American Psychiatric Publishing, Inc.

Glasser, M.F., Coalson, T.S., Robinson, E.C., Hacker, C.D., Harwell, J., Yacoub, E., Ugurbil, K., Andersson, J.L.R., Beckmann, C.F., Jenkinson, M., Smith, S.M., van Essen, D.C., 2016. A multi-modal parcellation of human cerebral cortex. Nature 536, 171–178. doi:10.1038/nature18933

Glasser, M.F., Sotiropoulos, S.N., Wilson, J.A., Coalson, T.S., Fischl, B., Andersson, J.L.R., Xu, J., Jbabdi, S., Webster, M., Polimeni, J.R., van Essen, D.C., Jenkinson, M., for the WU-Minn HCP Consortium, 2013. The minimal preprocessing pipelines for the Human Connectome Project. doi:10.1016/j.neuroimage.2013.04.127

Gordon, E.M., Laumann, T.O., Adeyemo, B., Huckins, J.F., Kelley, W.M., Petersen, S.E., 2014. Generation and Evaluation of a Cortical Area Parcellation from Resting-State Correlations. doi:10.1093/cercor/bhu239

Grinband, J., Steffener, J., Razlighi, Q.R., Stern, Y., 2017. BOLD neurovascular coupling does not change significantly with normal aging. Hum Brain Mapp. doi:10.1002/hbm.23608

Horiguchi, H., Wandell, B.A., Winawer, J., 2016. A Predominantly Visual Subdivision of The Right Temporo-Parietal Junction (vTPJ) 26, 639–646. doi:10.1093/cercor/bhu226

Jerde, T.A., Curtis, C.E., 2013. Maps of space in human frontoparietal cortex. J. Physiol. Paris 107, 510–516. doi:10.1016/j.jphysparis.2013.04.002

Jimenez, A.M., Lee, J., Wynn, J.K., Cohen, M.S., Engel, S.A., Glahn, D.C., Nuechterlein, K.H., Reavis, E.A., Green, M.F., 2016. Abnormal Ventral and Dorsal Attention Network Activity during Single and Dual Target Detection in Schizophrenia. Front Psychol 7, 323. doi:10.3389/fpsyg.2016.00323

Kay, S.R., Fiszbein, A., Opler, L.A., 1987. The positive and negative syndrome scale (PANSS) for schizophrenia. Schizophr Bull 13, 261–276.

Kubit, B., Jack, A.I., 2013. Rethinking the role of the rTPJ in attention and social cognition in light of the opposing domains hypothesis: findings from an ALE-based meta-analysis and resting-state functional connectivity. Front Hum Neurosci 7, 323. doi:10.3389/fnhum.2013.00323

Lomb, N.R., 1976. Least-squares frequency analysis of unequally spaced data. Astrophys Space Sci 39, 447–462. doi:10.1007/BF00648343

Ollinger, J.M., Corbetta, M., Shulman, G.L., 2001a. Separating processes within a trial in event-related functional MRI II. Analysis. NeuroImage 13, 218–229. doi:10.1006/nimg.2000.0711

Ollinger, J.M., Shulman, G.L., Corbetta, M., 2001b. Separating processes within a trial in event-related functional MRI I. The Method. NeuroImage 13, 210–217. doi:10.1006/nimg.2000.0710

Patel, G.H., Shulman, G.L., Baker, J.T., Akbudak, E., Snyder, A.Z., Snyder, L.H., Corbetta, M., 2010. Topographic organization of macaque area LIP. Proc Natl Acad Sci USA 107, 4728–4733. doi:10.1073/pnas.0908092107

Patel, G.H., Yang, D., Jamerson, E.C., Snyder, L.H., Corbetta, M., Ferrera, V.P., 2015. Functional evolution of new and expanded attention networks in humans. Proc Natl Acad Sci USA. doi:10.1073/pnas.1420395112

Pelli, D.G., 1997. The VideoToolbox software for visual psychophysics: transforming numbers into movies. Spatial Vision 10, 437–442. doi:10.1163/156856897X00366

Power, J.D., 2016. A simple but useful way to assess fMRI scan qualities. doi:10.1016/j.neuroimage.2016.08.009

Power, J.D., Mitra, A., Laumann, T.O., Snyder, A.Z., Schlaggar, B.L., Petersen, S.E., 2014. Methods to detect, characterize, and remove motion artifact in resting state fMRI. 84, 320–341. doi:10.1016/j.neuroimage.2013.08.048

Shenhav, A., Cohen, J.D., Botvinick, M.M., 2016. Dorsal anterior cingulate cortex and the value of control. Nature Publishing Group 19, 1286–1291. doi:10.1038/nn.4384

Shulman, G.L., McAvoy, M.P., Cowan, M.C., Astafiev, S.V., Tansy, A.P., d'Avossa, G., Corbetta, M., 2003. Quantitative analysis of attention and detection signals during visual search. J Neurophysiol 90, 3384–3397. doi:10.1152/jn.00343.2003

Silver, M.A., Kastner, S., 2009. Topographic maps in human frontal and parietal cortex. Trends Cogn Sci (Regul Ed) 13, 488–495. doi:10.1016/j.tics.2009.08.005

Squire, R.F., Noudoost, B., Schafer, R.J., Moore, T., 2013. Prefrontal contributions to visual selective attention. Annu Rev Neurosci 36, 451–466. doi:10.1146/annurev-neuro-062111-150439

van Essen, D.C., Glasser, M.F., Dierker, D.L., Harwell, J., Coalson, T., 2012. Parcellations and hemispheric asymmetries of human cerebral cortex analyzed on surface-based atlases. 22, 2241–2262. doi:10.1093/cercor/bhr291

Van Snellenberg, J.X., 2009. Working memory and long-term memory deficits in schizophrenia: is there a common substrate? Psychiatry research 174, 89–96. doi:10.1016/j.pscychresns.2009.04.001

Whitfield-Gabrieli, S., Ford, J.M., 2012. Default Mode Network Activity and Connectivity in Psychopathology. http://dx.doi.org/10.1146/annurev-clinpsy-032511-143049 8, 49–76. doi:10.1146/annurev-clinpsy-032511-143049

Wynn, J.K., Jimenez, A.M., Roach, B.J., Korb, A., Lee, J., Horan, W.P., Ford, J.M., Green, M.F., 2015. Impaired target detection in schizophrenia and the ventral attentional network: Findings from a joint event-related potential-functional MRI analysis. YNICL 9, 95–102. doi:10.1016/j.nicl.2015.07.004

Yeo, B.T.T., Krienen, F.M., Sepulcre, J., Sabuncu, M.R., Lashkari, D., Hollinshead, M., Roffman, J.L., Smoller, J.W., Zollei, L., Polimeni, J.R., Fischl, B., Liu, H., Buckner, R.L., 2011. The organization of the human cerebral cortex estimated by intrinsic functional connectivity. J Neurophysiol 106, 1125–1165. doi:10.1152/jn.00338.2011
