# Supplementary material for: Deficits and compensation: Attentional control cortical networks in schizophrenia
Source: Neuroimage Clin. 2020 Jul 20;27:102348. doi: 10.1016/j.nicl.2020.102348 (PMC7393326; doi:10.1016/j.nicl.2020.102348)
Supplement: Supplementary data 2 [file mmc2.docx]

**Supplemental Table 1**

| Functionally defined ROIs | Attentional Control Component | Human Connectome Project Parcellation Scheme (Glasser et al. 2016) | Gordon et al. 2014 | Other Parcellation Schemes |
| --- | --- | --- | --- | --- |
| R/L V1 | Visual | R/L V1 | R Visual 21; L Visual 3 |  |
| R/L V23d | Visual | R/L V2, V3 | R Visual 35; - |  |
| R/L V23v | Visual | R/L V2, V3 | R Visual 37; L Visual 18 |  |
| R/L V3B | Visual | R/L V3B, IPO, V3 C/D | R Visual 27; L Visual 1 |  |
| R/L V4 | Visual | R/L ventral aspects of V4 | R Visual 33; L Visual 18 |  |
| R/L V4t | Visual | R/L V4t, LO2, PIT | R Visual 29; L Visual 16, L Visual 10 |  |
| R/L VOT | Visual | R/L V8, VVC, VMV3, FFC | R Visual 33, R Visual 34; L Visual 11, L Visual 12, L Visual 13 |  |
| R/L PH | Visual | R/L PH, FFC | R Visual 30; L Visual 16 |  |
| R/L MT | Visual | R/L MT, MST | R Visual 29; L Visual 10 |  |
| R/L LO | Visual | R/L V3C/D, V4, LO1, LO3, PGp | R Visual 28; L Visual 8 |  |
| R/L vIPS 1 | Dorsal Attention | R/L V7 | R Dorsal Attention 28; L Dorsal Attention 10 | V7^1^ |
| R/L vIPS 2 | Dorsal Attention | R/L IPS1 | R Dorsal Attention 27; L Dorsal Attention 9 | IPS 1^1^ |
| R/L pIPS | Dorsal Attention | R/L LIPv, LIPd, VIP | R Visual 24; - | IPS 2/3/4^3^, IPS 2/3^4^ |
| R/L aIPS | Dorsal Attention | R/L posterior aspect of AIP | R Dorsal Attention 24, R Dorsal Attention 29; L Dorsal Attention 5 | IPS 5^3^ |
| R/L pSTG | Dorsal Attention | R/L PSL | R Visual 24; - | STG^2^ |
| R/L FEF | Dorsal Attention | R/L FEF | R Dorsal Attention 21, R Dorsal Attention 22, R Cingulo-Opercular 28; L Dorsal Attention 1, L Dorsal Attention 2, L Cingulo-Opercular 6, L Somatomotor mouth 2 |  |
| R/L iPCS 1 | Dorsal Attention | R/L PEF | R Dorsal Attention 31; L Dorsal Attention 15 |  |
| R/L iPCS 2 | Lateral PFC | R/L IFJp | R Dorsal Attention 32; L Dorsal Attention 15, L Dorsal Attention 16, L Dorsal Attention 18 |  |
| R/L aIFS | Lateral PFC | R/L 9-46v, IFSp, IFSa | R Frontoparietal 18; L Dorsal Attention 8, L Dorsal Attention 18, L Frontoparietal 7 |  |
| R/L pIFS | Lateral PFC | R/L IFJa, L IFSp | R Frontoparietal 19; - | IFJa^5^ |
| R/L aIns1 | Cingulo-Opercular/Salience | R/L FOP4, FOP5 | R Cingulo-Opercular 37; L Cingulo-Opercular 13 |  |
| R/L aIns2 | Cingulo-Opercular/Salience | R/L AVI | R Cingulo-Opercular 36, R Cingulo-Opercular 37, R Salience 4; L Cingulo-Opercular 13, L Salience 2 |  |
| R/L dACC | Cingulo-Opercular/Salience | R/L 8BM, SCEF | R Cingulo-Opercular 22, R Cingulo-Opercular 24; L Frontoparietal 3 |  |
| R a32 | Cingulo-Opercular/Salience | R a32 PR, d32, p23PR | -; L Cingulo-Opercular 4, L Salience 1 |  |
| R TPJ | Ventral Attention | R PSL, R STV, R PFm | R Cingulo-Opercular 29 |  |
| R VFC | Ventral Attention | R FOP3 | R Cingulo-Opercular 33 |  |

**Supplementary Table 1:** This table shows how the RSVP functionally defined ROIs map onto previously published parcellation schemes and studies.
