# Supplementary material for: Deficits and compensation: Attentional control cortical networks in schizophrenia
Source: Neuroimage Clin. 2020 Jul 20;27:102348. doi: 10.1016/j.nicl.2020.102348 (PMC7393326; doi:10.1016/j.nicl.2020.102348)
Supplement: Supplementary data 3 [file mmc3.docx]

**Supplementary Table 2**

| **MCCB Domains** | **T-score** |
| --- | --- |
| Speed of Processing | 41.22 |
| Attention/Vigilance | 42.00 |
| Working Memory | 41.67 |
| Verbal Learning | 42.50 |
| Visual Learning | 40.31 |
| Reasoning Problem Solving | 42.56 |

**Supplementary Table 2:** This table shows the average T-score for each cognitive domain of the MATRICS Cognitive Consensus Battery (MCCB) (T-scores age- and gender-corrected).
